# Effects of timing of abomasal infusion of fatty acids on the daily rhythms of milk synthesis and plasma hormones and metabolites in dairy cows

**DOI:** 10.3168/jds.2024-26215

**Published:** 2025-05-16

**Authors:** I. J. Salfer, P. A. Bartell, K. J. Harvatine

**Affiliations:** Department of Animal Science, The Pennsylvania State University, University Park, PA 16802

**Keywords:** daily rhythm, milk synthesis, nutrient entrainment, circadian rhythm

## Abstract

Dairy cows display daily rhythms of milk synthesis that appear to be driven by a circadian clock located in the mammary gland. These rhythms are altered by the time of feed availability. Fatty acids have been shown to entrain circadian rhythms in liver and adipose tissue in experimental models, but their role in the mammary gland has not been well investigated. Our objective was to determine the effects of the timing of fatty acid absorption on the daily rhythms of milk synthesis. Nine lactating Holstein cows were arranged in a 3 × 3 Latin square design. Treatments were abomasal infusions of 350 g/d of a free fatty acid stock enriched in *cis*-9 18:1 either continuously throughout the day for 22 h (CON) or for 8 h from 0900 to 1700 h (DAY) or from 2100 to 0500 h (NGT). Treatment periods were 12 d with a 5-d washout. Cows were milked every 6 h during the final 7 d of each period to determine the daily patterns of milk synthesis. A 24-h rhythm was fit to time course data using cosine analysis, and the amplitude and acrophase (time at peak) were determined. Daily milk and milk protein yields were decreased by DAY and NGT compared with CON, whereas milk fat yield was not changed. Milk yield fit a 24-h rhythm in CON and DAY but not in NGT. Furthermore, DAY delayed the peak of the daily rhythm of milk yield by 2 h compared with CON. Fat and protein concentrations exhibited daily rhythms in CON and NGT but not DAY. Fat yield only fit a 24-h rhythm in DAY. Both de novo and mixed-source fatty acid yields were reduced by DAY and NGT, suggesting that the faster infusion rates may have resulted in concentrations of fatty acids that exceeded a threshold sufficient to inhibit de novo fatty acid synthesis. Plasma glucose concentration failed to display a daily rhythm in any treatment, whereas nonesterified fatty acids showed a rhythm in CON and NGT, but this rhythm was abolished by DAY. Insulin fit a rhythm in NGT and tended to fit a rhythm with a lower amplitude in CON, but no rhythm was present in DAY. Blood urea nitrogen exhibited a daily rhythm under all treatments, and both the mean and amplitude were increased by DAY. Daily rhythms of milk synthesis were also modified by DAY, with a slight delay in the daily peak of milk yield and elimination of the rhythms of milk fat and protein concentrations. Infusion at night had little effect. Daytime infusion also modified the daily rhythms of plasma metabolites by reducing the amplitude of nonesterified fatty acid concentration and increased the amplitude of blood urea nitrogen.

## INTRODUCTION

Dairy cows exhibit a daily pattern of feed intake with high rates of intake during the morning and afternoon and a decline in intake overnight (Albright, 1993). Furthermore, intake is stimulated by delivery of fresh feed and milking (Menzi and Chase, 1994; DeVries et al., 2005). Although most cows in the United States are fed a TMR, which provides a consistent composition of nutrients over the day, the daily pattern of feed intake causes the consumption of nutrients to vary across the day (Ying et al., 2015). The daily pattern of feed intake can be altered by modifying the time of feed delivery (Niu et al., 2014), the frequency of feeding (Rottman et al., 2014), and the frequency of milking (Hart et al., 2013).

In addition to a daily pattern of feed intake, cows display daily rhythms of milk and milk component synthesis. In herds feeding a complete ration 1×/d in the morning, milk yield is greatest in the morning, and milk fat and protein concentration are greatest in the evening (Rottman et al., 2014; Ying et al., 2015). However, night-restricted feeding shifts these rhythms so that milk yield peaks in the evening and milk fat and protein concentrations peak in the morning (Salfer and Harvatine, 2020)

Circadian rhythms are generated in both the brain and peripheral tissues by “clock” transcription factors that oscillate over 24 h and can be entrained by environmental factors. Light is the primary signal that entrains central circadian rhythms, but an increasing body of evidence has demonstrated a role for timing of food intake in the circadian entrainment of peripheral tissues. Fatty acids (**FA**), in particular, play an important role in entrainment of circadian rhythms in many different model organisms. In mice and rats, feeding a high-fat diet desynchronizes the circadian rhythm of activity from the light-dark cycle and dampens the rhythms of clock gene expression in the hypothalamus, liver, and adipose tissue (Bartol-Munier et al., 2006; Kohsaka et al., 2007).

Although feed intake entrains the daily rhythms of milk synthesis, the roles of individual nutrients, such as FA, are unknown. The objective of this experiment was to determine the effects of the timing of FA absorption on the daily rhythms of milk synthesis, plasma metabolites, and body temperature. We hypothesize that infusing FA into the abomasum over 8 h during the day or 8 h during the night would shift the phase of the daily rhythms of milk fat synthesis relative to the phases of these rhythms under continuous infusion across the day. Furthermore, we expect the daily rhythms of plasma nonesterified FA (**NEFA**) concentration and core body temperature to be shifted by the timing of FA infusion. Companion studies also investigated the effects of the timing of abomasal infusion of sodium caseinate (Salfer et al., 2023) and ruminal infusion of acetate (Matamoros et al., 2022) on daily rhythms of milk synthesis and plasma metabolites.

## MATERIALS AND METHODS

### Animals and Treatments

Nine multiparous, rumen-cannulated, mid-lactation (132 ± 90 d postpartum; mean ± SD) Holstein cows from the Penn State University Dairy Research and Teaching Center were randomly assigned to treatment sequences in a 3 × 3 Latin square design, with 6 balanced sequences and an additional 3 sequences randomly selected from the set of 6 balanced sequences. Sample size was determined based on a >80% power of observing *P* < 0.05 for a 2.5-kg difference in milk yield based on variance observed in previous experiments (Niu et al., 2014; Rottman et al., 2014). Treatment periods were 12 d, with 10 d of treatment adaptation and 2 d of sampling. There was a 5-d washout between periods.

**Figure 1.**
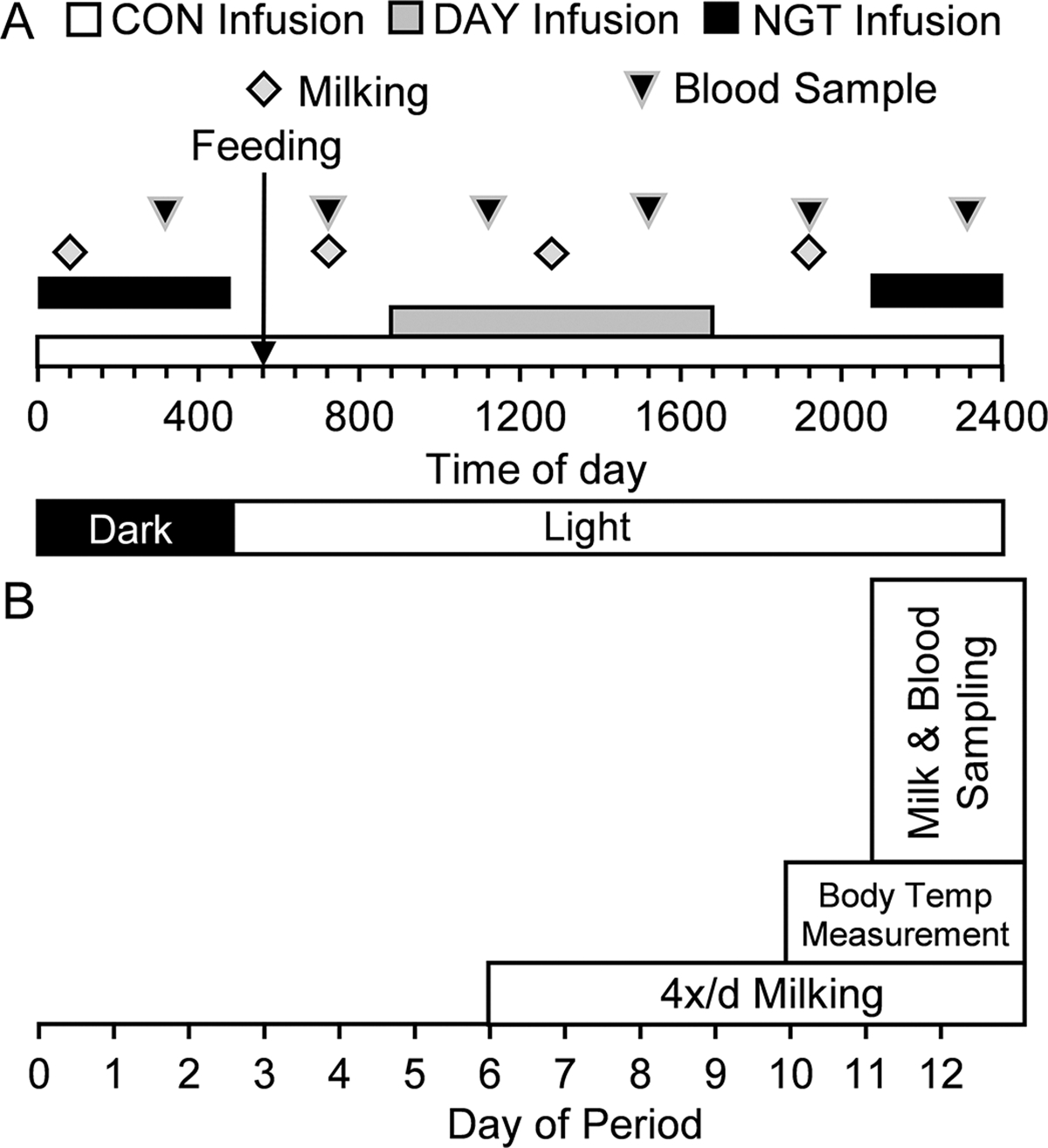
Schedule of feeding, lighting, and sampling during the experiment. (A) Daily experimental schedules of treatments and sample collections during the sampling period. Feeding was conducted at 0600 h; milking was performed at 0100, 0700, 1300, and 1900 h; and blood was collected at 0030, 0430, 0830, 1230, 1630, and 2030 h. Treatments were 350 g/d of free fatty acids abomasally infused continuously for 22 h/d (CON), or for 8 h/d during the day (DAY; 0900–1700 h) or the night (NGT; 2100–0500 h). (B) Schedule of experimental periods. Each period was a total of 12 d, with 7 d of adaptation to infusion, 7 d of adaptation to 4×/d milking, 3 d of body temperature (temp) measurement, and 2 d of sampling. Three experimental periods were used, with a 3 × 3 Latin square organization of treatments.

Treatments were 350 g/d of a free FA stock abomasally infused either continuously for 22 h/d (**CON**); for 8 h/d from 0900 h to 1700 h (**DAY**); or for 8 h/d from 2100 h to 0500 h (**NGT**; Figure 1). The DAY and NGT treatments were designed to represent the highest and lowest feed intake periods of the day (DeVries et al., 2005), and CON was designed to be iso-FA and represent a consistent amount of available substrate throughout the day. The free FA stock contained 5.1% 16:0, 1.4% 18:0, 79.6% *cis*-9 18:1, and 10.0% 18:2 n-6 (KIC Chemicals Inc., New Paltz, NY; Table 1), and 10% wt/wt Tween 80 was added to aid emulsification. The infusion dose was selected to represent approximately a 1.5 percentage-unit increase in dietary fat and ~50% of daily milk preformed FA yield. Cows in the CON treatment were infused continuously throughout the day except for 30 min during each milking. The experiment was conducted from May 7 to June 22, 2018. All experimental procedures were approved by the Penn State University Institutional Care and Use Committee.

### Feed Intake and Feeding Behavior

All cows were provided the same basal TMR fed once daily at 0600 h at 110% of the previous day’s intake (Table 2). Feed samples were collected on d 7 of each period and analyzed for DM, ash, NDF, ADF, starch, and CP, according to (Rico and Harvatine, 2013). The daily pattern of feed intake was monitored using an automated feed observation system described by (Rottman et al., 2015). Briefly, hanging feed tubs were suspended from electronic load cells connected to an electronic data acquisition system, which measured and recorded feed weight every 10 s from d 8 to 12 of each period. Feed weight data were smoothed by first calculating a running averaging over 180 s, and the rate of feed intake across the day was determined over 10-min intervals and subsequently averaged into 2-h intervals. The number, length, and size of meals, intermeal interval, eating time, and eating rate were determined as described by (Niu et al., 2017) using a minimal intermeal interval of 8 min.

### Milk Sampling and Analysis

Cows were milked every 6 h during the final 7 d of each period (0100, 0700, 1300, and 1900 h) to observe the daily rhythm of milk synthesis. Milk collected at each time point represented the sum of milk synthesis over the previous 6-h interval and is plotted as the midpoint of the milking interval (3 h before collection). Milk yield was measured at each milking using an integrated milk meter (AfiMilk MPC Milk Meter, Afimilk Agricultural Cooperative Ltd., Kibbutz Afikim, Israel). Yields were corrected for the deviation of each individual stall according to Rico and Harvatine (2013). Milk was sampled at each milking during the final 2 d of each period. One aliquot was stored at 4°C with preservative (Bronolab-WII, Advanced Instruments Inc., Noorwood, MA) before analysis for fat and protein concentrations by Fourier-transform infrared spectroscopy (Fossomatic 4000 Milko-Scan and 400 Fossomatic, Foss Electric; analysis conducted by Dairy One DHIA, Ithaca, NY). A second aliquot was stored at 4°C and centrifuged at 2,300 × *g* at 4°C for 20 min within 24 h. The resulting fat cakes were stored at −20°C and analyzed for concentrations of individual FA according to Andreen et al. (2020). Milk FA originating from de novo synthesis in the mammary gland (Σ even straight-chain FA <16C), originating from both de novo synthesis and plasma FA uptake (Σ straight-chain 16C), and originating exclusively from plasma (Σ even straight-chain FA >16C) were calculated (Bauman and Griinari, 2003).

### Plasma Sampling and Analysis

Blood was sampled via venipuncture of a coccygeal vessel into potassium-EDTA vacuum tubes (BD Vacutainer, Becton Dickinson, Franklin Lakes, NJ) at 6 times from d 11 to 12 to represent every 4 h across the day (0300, 0700, 1100, 1500, 1900, and 2300 h). Samples were immediately placed on ice and centrifuged within 30 min at 2,300 × *g* for 15 min at 4°C. Plasma was collected and stored at −20°C for analysis of glucose, nonesterified FA, and BUN as described by (Rottman et al., 2014). Briefly, plasma glucose concentration was analyzed using a glucose oxidase/peroxidase enzymatic colorimetric assay (No. P 7119, Sigma-Aldrich, St. Louis, MO), NEFA concentration was measured using an acyl-CoA oxidase/peroxidase enzymatic colorimetric assay (NEFA-HR 2, Wako Diagnostics, Richmond, VA), and BUN was assayed using a modified Berthelot methodology (Modified Enzymatic Urea Nitrogen Procedure No. 2050, Stanbio Laboratory, Boerne, TX). Insulin was measured using a porcine ^125^I-insulin radioimmunoassay kit with correction based on a bovine insulin standard (PI-12 K Porcine Insulin RIA, EMD Millipore).

### Body Temperature Analysis

An intravaginal temperature logger was used to record core body temperature every 10 min on d 10 to 12 of each period as described by (Niu et al., 2017). Briefly, a miniature plastic-coated thermometer fastened to a hormone-free vaginal implant (progesterone-free CIDR, Zoetis Inc., Parsippany-Troy Hills, NJ) was placed centrally in the vagina. Body temperature was averaged over 2-h intervals.

### Statistical Analysis

All data were analyzed using the MIXED procedure of SAS 9.4 (SAS Institute Inc., Cary, NC). Models testing the effects of treatment on daily parameters, including DMI, milk production, and FA yields, included the fixed effect of treatment and the random effects of cow and period. Individual treatment LSM were compared using preplanned contrasts of CON versus DAY, CON versus NGT, and DAY versus NGT.

The linear effects of treatment, time, and their interaction were first analyzed by a mixed effects model that included the random effects of cow and period. Cow by period was the subject, the first-order autoregressive or first-order autoregressive heterogeneous covariance structure was selected based on convergence criteria, and denominator degrees of freedom were adjusted using the Kenward-Roger method. Second, for responses that were significantly affected by time, the fit, amplitude, and acrophase of a 24-h rhythm of milk yield and components, FA profile, plasma hormones and metabolites, and body temperature were determined using cosinor rhythmometry using mixed model random regression to fit the data to the linear form of cosine functions with a period of 24 h in SAS 9.4, as described by (Rottman et al., 2014). The model included the fixed effects of treatment and cosine parameters, the interactions between treatment and cosine parameters, and the random effects of cow and period. The fit of the 24-h cosine curve was determined using a zero-amplitude test, and the amplitude and acrophase (time at peak of rhythm) were determined according to Salfer et al. (2019). In all analyses, outliers with Studentized residuals outside ±3.5 (1 out of 144, 1 out of 144, 3 out of 150, and 7 out of 150 individual data points for the fat concentration rhythm, the protein concentration rhythm, the insulin concentration rhythm, and the NEFA concentration rhythm, respectively) were removed. A separate model testing the fixed effects of treatment, time, and their interaction, with random effects of cow and period, and preplanned contrasts testing the interaction of treatment and time were also conducted on all time course data. Statistical significance was declared at *P* < 0.05 and trends acknowledged at 0.05 < *P* < 0.10. High-resolution figures were generated using an add-in for Microsoft Excel (Kraus, 2014).

## RESULTS

### Daily Milk and Milk Component Synthesis

**Figure 2.**
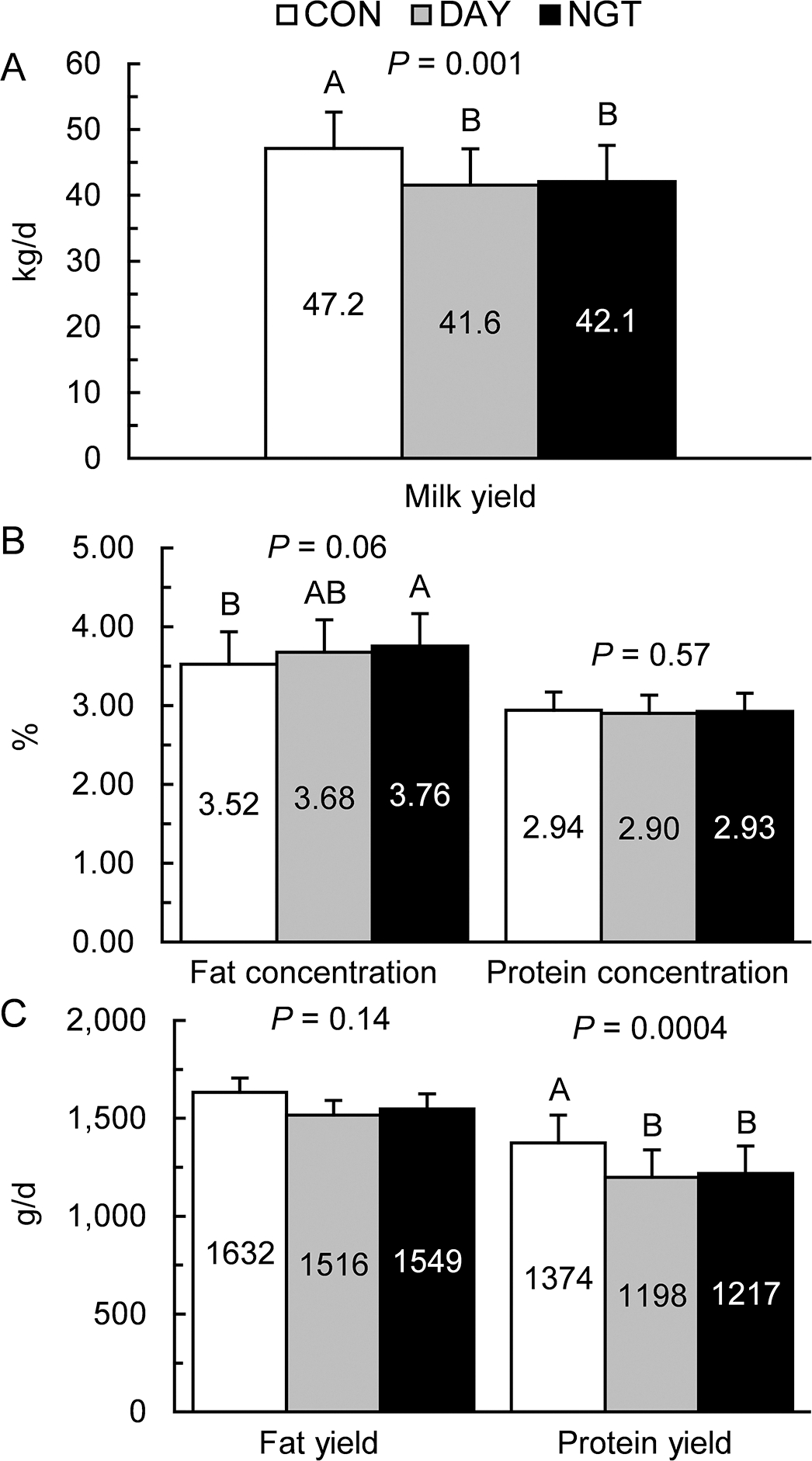
Effects of the timing of fatty acid (FA) infusion on daily milk, fat, and protein yields, and fat and protein concentrations. Treatments were 350 g/d of free FA abomasally infused continuously for 22 h/d (CON), or for 8 h/d during the day (DAY; 0900–1700 h) or the night (NGT; 2100–0500 h). Panels show the effects of time of FA infusion on (A) milk yield (kg), (B) fat and protein concentrations (%), and (C) fat and protein yields (g). Data are presented as LSM with SEM bars. Means that do not share a letter differ (*P* < 0.05).

Abomasal infusion of the FA solution during DAY and NGT decreased milk yield by 12% and 11%, respectively (*P* = 0.001; Figure 2), compared with continuous infusions. The time of FA infusion did not change milk fat yield (*P* = 0.14) but tended to alter milk fat concentration (*P* = 0.06), with NGT increasing milk fat percent 0.24 percentage units compared with CON (*P* = 0.02). Milk protein yield was decreased 13% in DAY and 11% in NGT compared with CON (*P* = 0.0004), but milk protein concentration was not affected by treatment (*P* = 0.57).

### Daily Rhythms of Milk Yield and Components

**Figure 3.**
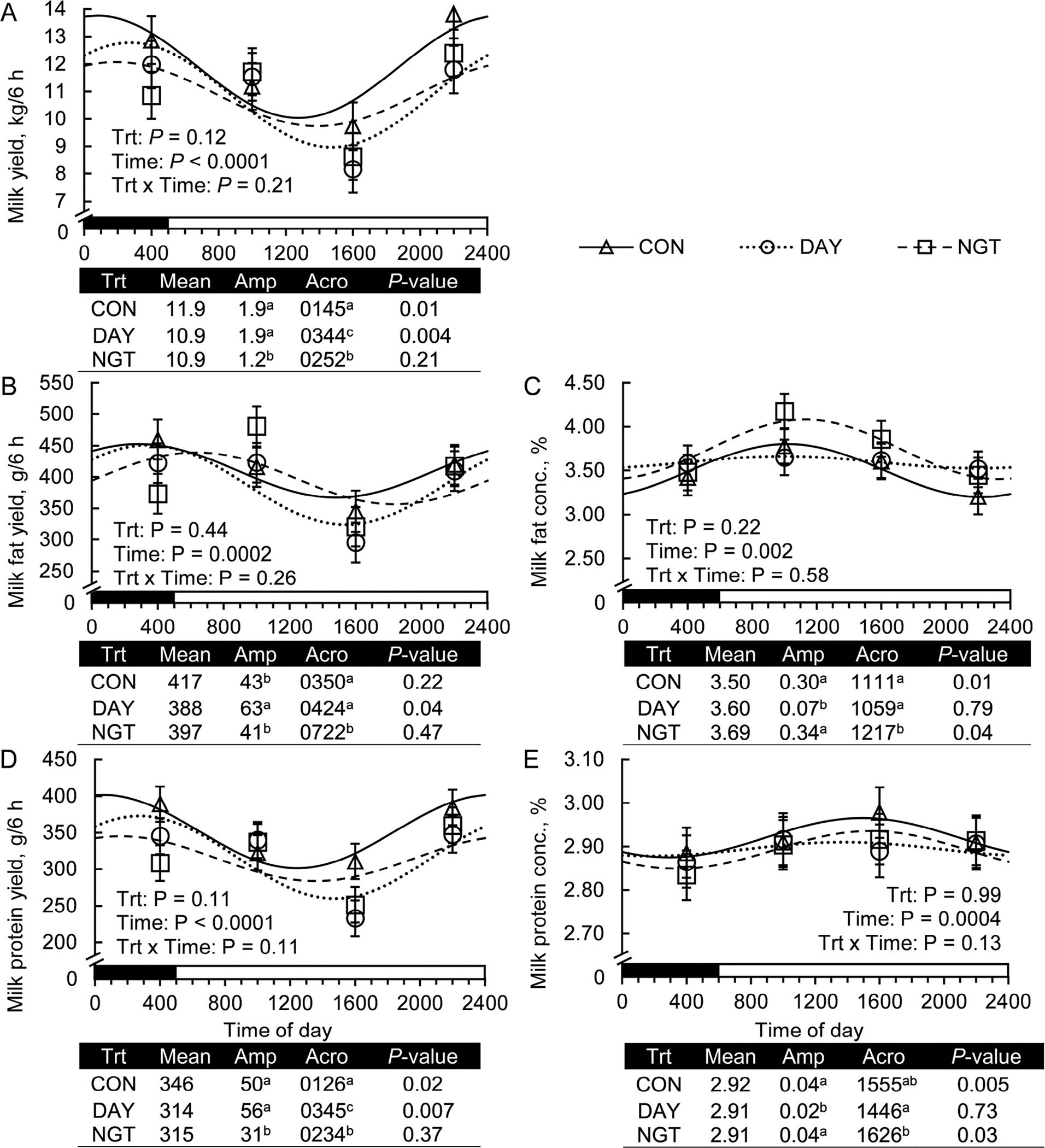
Effects of the timing of fatty acid (FA) infusion on the daily rhythms of milk synthesis. Treatments (Trt) were 350 g/d of free FA abomasally infused continuously for 22 h/d (CON), or for 8 h/d during the day (DAY; 0900–1700 h) or the night (NGT; 2100–0500 h). Milk was collected every 6 h across the day (0100, 0700, 1300, and 1900 h), and data are presented at the midpoint of each milking interval (0400, 1000, 1600, and 2200 h). Panels show the effects of time of FA infusion on the daily rhythms of milk (A) yield, (B) fat yield, (C) fat concentration (conc.), (D) protein yield, and (E) protein concentration. Data are presented as relative expression LSM with SEM bars and fitted cosine curve. Cosinor analysis output is shown below each panel and includes the amplitude (Amp; difference between peak and mean), acrophase (Acro; time at peak of the rhythm), and *P*-value of the zero-amplitude test. Means that do not share a superscript (a, b, or c) differ (*P* < 0.05). The black and white bars above the x-axis display the light:dark cycle.

Milk yield exhibited a 24-h rhythm when FA were infused either continuously (*P* = 0.01) or only during the day (*P* = 0.004), but not when infused only at night (*P* = 0.21; Figure 3A). The amplitude of the rhythm of milk yield did not differ between CON and DAY but was reduced 37% by NGT (*P* < 0.05). Moreover, DAY and NGT delayed the phase of the daily rhythm compared with CON by 2 h and 1.1 h, respectively, as indicated by differences in the acrophase (*P* < 0.05). Milk fat concentration followed a 24-h rhythm in CON (*P* = 0.01) and NGT (*P* = 0.04) but not in DAY (*P* = 0.79). No difference in amplitude was found between CON and NGT (*P* > 0.10; Figure 3C). The peak of the rhythm of milk fat concentration was shifted 1.1 h in NGT compared with CON. A 24-h rhythm of milk protein concentration was also present in CON (*P* = 0.005) and NGT (*P* = 0.03) but not DAY (*P* = 0.73; Figure 3E). As with milk fat concentration, there was no difference in rhythm amplitude between CON and NGT (*P* > 0.10). Furthermore, the amplitude of the 24-h rhythm of milk fat concentration was not different between CON and NGT (*P* > 0.05).

Milk fat yield fit a daily rhythm in DAY (*P* = 0.04) but not CON (*P* = 0.22) or NGT (*P* = 0.47; Figure 3B). The rhythm of DAY peaked at 0424 h and had an amplitude of 63 g/6 h. A 24-h rhythm fit the daily pattern of milk protein yield in CON (*P* = 0.02) and DAY (*P* = 0.007), but not NGT (*P* = 0.37; Figure 3D). The amplitude of the daily rhythm did not differ between CON and DAY (*P* > 0.10), but the time of acrophase was delayed 2.3 h by DAY compared with CON (*P* < 0.05).

### Daily Yields and Rhythms of Milk FA

**Figure 4.**
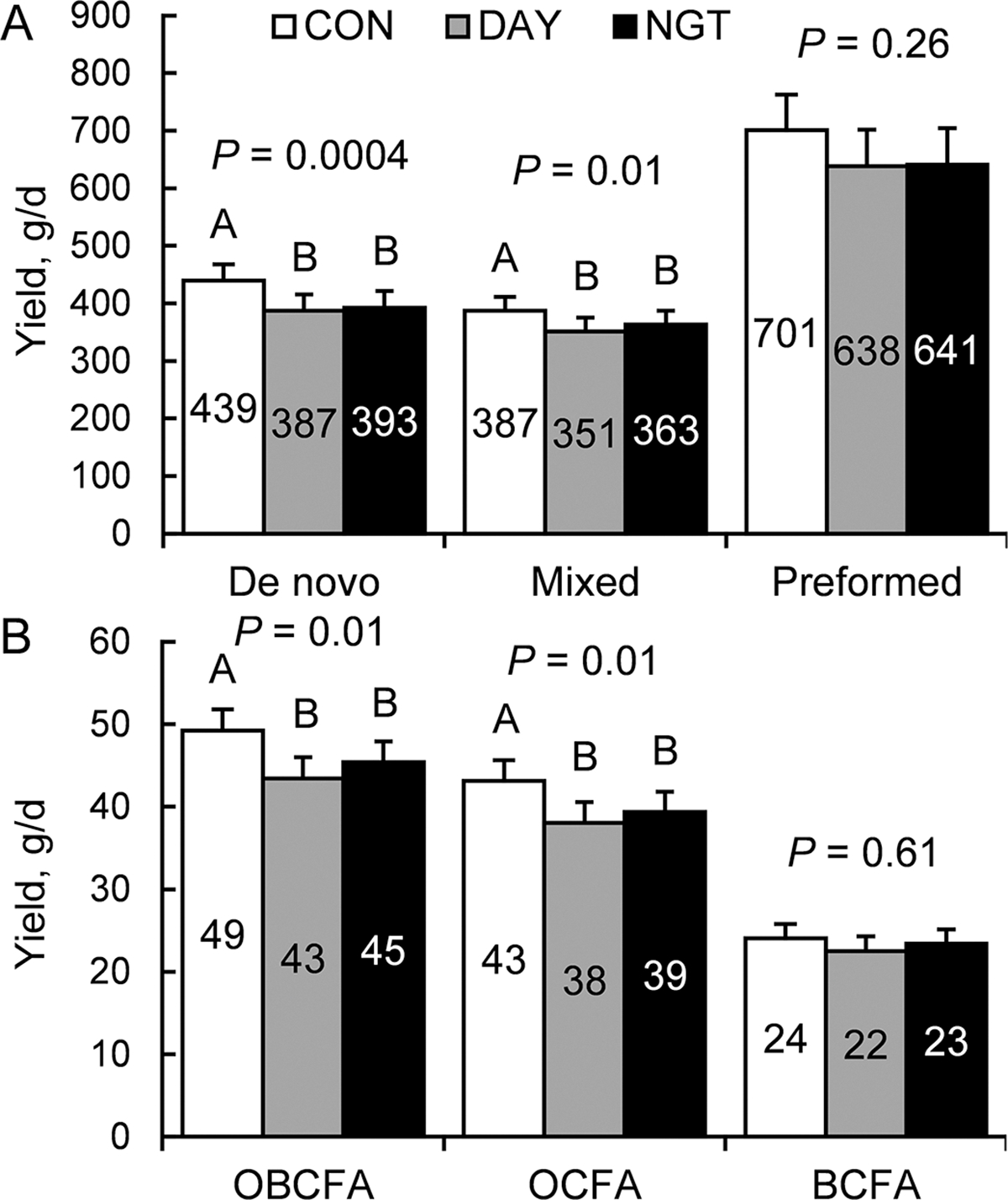
Effects of the timing of fatty acid (FA) infusion on the daily yields of milk FA. Treatments were 350 g/d of free FA abomasally infused continuously for 22 h/d (CON), or for 8 h/d during the day (DAY; 0900–1700 h) or the night (NGT; 2100–0500 h). Panels show the effects of time of FA infusion on the average daily yields of (A) de novo (Σ <16C), mixed (Σ 16C), and preformed (Σ >16C) sources of FA (g/d), and (B) odd- and branched-chain FA (OBCFA), odd-chain FA (OCFA), and branched-chain FA (BCFA; g/d). Data are presented as LSM with SEM bars.

In addition to examining total fat yield, the effects of the timing of FA infusion on the synthesis of milk FA was examined. The yield of FA originating from de novo synthesis in the mammary gland (Σ < 16C) was affected by treatment (*P* = 0.0004), with DAY and NGT reducing yields by 12% and 9%, respectively, compared with CON (*P* < 0.0005; Figure 4A). Similarly, yields of mixed-source FA originating both from de novo synthesis and the uptake of preformed FA from plasma (Σ 16C) were decreased 9% by DAY and 6% by NGT compared with CON (*P* = 0.01). Yields of preformed FA (Σ > 16C), however, were not affected by treatment (*P* = 0.26).

Of the individual types of FA, 3 odd-chain FA (**OCFA**) emerged as having the greatest decreases in yield from CON in both DAY and NGT treatments (*P* < 0.02; Figure 4B). These included 11:0 (28% decrease in DAY; 27% decrease in NGT; *P* = 0.03), 13:0 (28% decrease in DAY; 23% decrease in NGT; *P* = 0.002), and 15:0 (23% decrease in DAY; 16% decrease in NGT; *P* = 0.0004; Supplemental Table S1, see Notes). Total OCFA were reduced by 12% in DAY and 9% in NGT, but total odd- and branched-chain FA (**OBCFA**) were reduced by 12% in DAY and 8% in NGT. There was no difference in the abundance of branched-chain FA among treatments (*P* = 0.61).

**Figure 5.**
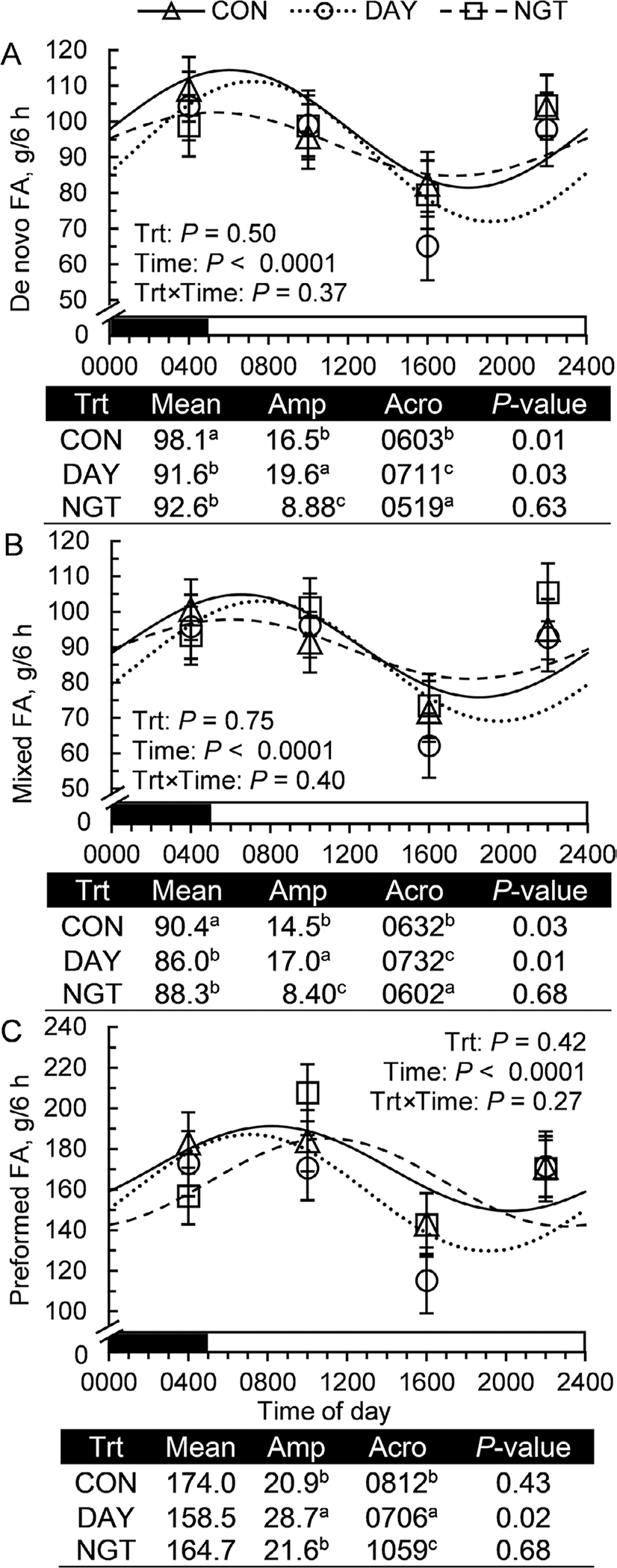
Effects of the timing of fatty acid (FA) infusion on the daily rhythms of milk FA. Treatments were 350 g/d of free FA abomasally-infused continuously for 22 h/d (CON), or for 8 h/d during the day (DAY; 0900–1700 h) or the night (NGT; 2100–0500 h). Panels show the effects of time of FA infusion on the daily rhythms of milk (A) de novo (Σ <16C), (B) mixed (Σ 16C), and (C) preformed (Σ >16C) yields. Data are presented as LSM with SEM bars. Cosinor analysis output is shown below each panel and includes the amplitude (Amp; difference between peak and mean), acrophase (Acro; time at peak of the rhythm), and *P*-value of the zero-amplitude test. Means with differing superscripts (a–c) are different at *P* < 0.05.

Daily rhythms of de novo, mixed, and preformed FA were also determined. De novo-synthesized FA followed a 24-h cosine function in CON (*P* = 0.01) and DAY (*P* = 0.03) but not NGT (*P* = 0.63), with a 19% greater amplitude in DAY than NGT (*P* < 0.05; Figure 5A). The rhythm of de novo FA peaked at 0603 h in CON and was shifted 1.1 h later in DAY (*P* < 0.05). Similarly, mixed-source FA fit a 24-h rhythm in CON (*P* = 0.03) and DAY (*P* = 0.01) but not NGT (*P* = 0.68), with a 17% greater amplitude and a 1-h phase delay in DAY compared with CON (*P* < 0.05; Figure 5B). Finally, preformed FA failed to fit a daily rhythm in CON (*P* = 0.43) or NGT (*P* = 0.68), but a rhythm with an amplitude of 28.7 g and an acrophase of 0706 h was induced in DAY (*P* = 0.02).

### Feed Intake and Feeding Behavior

**Figure 6.**
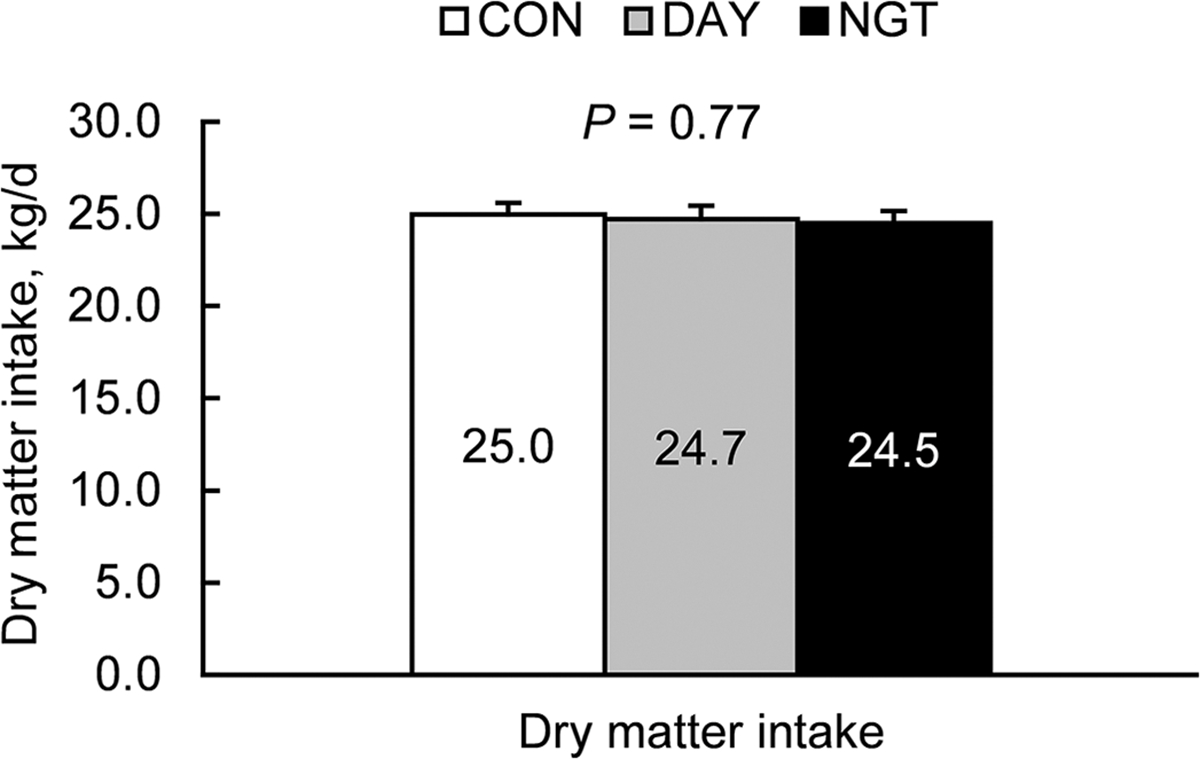
Effects of the timing of fatty acid (FA) infusion on DMI. Treatments were 350 g/d of free FA abomasally infused continuously for 22 h/d (CON), or for 8 h/d during the day (DAY; 0900–1700 h) or the night (NGT; 2100–0500 h). Data are presented as LSM with SEM bars.

**Figure 7.**
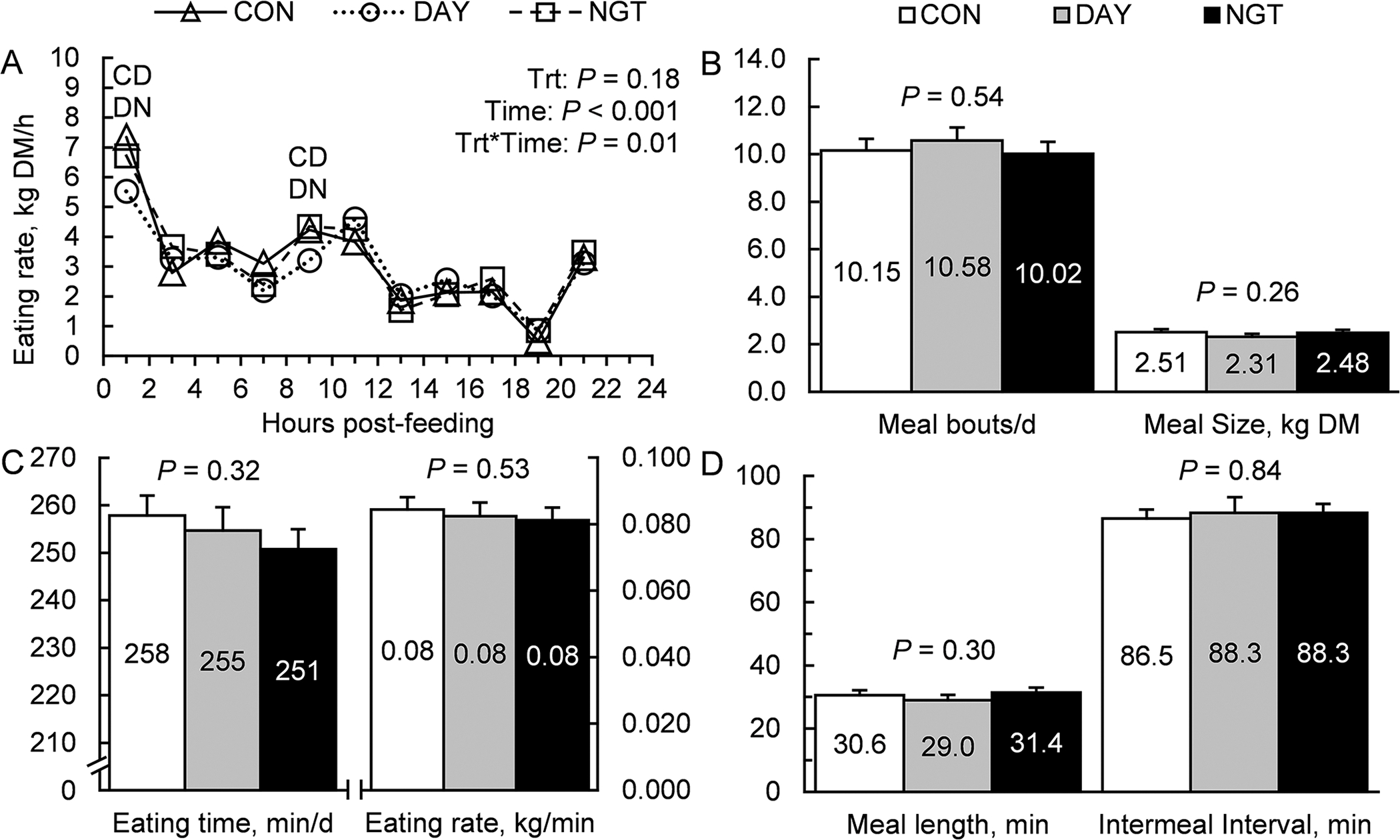
Effects of the timing of fatty acid (FA) infusions on feeding behavior. Treatments were 350 g/d of free FA abomasally infused continuously for 22 h/d (CON), or for 8 h/d during the day (DAY; 0900–1700 h) or the night (NGT; 2100–0500 h). Data are presented as LSM with SEM bars. Panels show the effects of time of FA infusion on (A) eating rate, (B) meal bouts/d and meal size, (C) eating time (min/d) and eating rate (kg/min), and (D) meal length and intermeal interval.

Total DMI was not affected by the timing of FA infusion (*P* = 0.77; Figure 6). However, treatments modified the daily pattern of feed intake, indicating an interaction in the effect of treatment and time (*P* = 0.01; Figure 7A). Specifically, CON and NGT had greater feed intake than DAY during both the first 2 h after feeding and at 8 h after feeding. Treatment did not affect the number of meal bouts per day (*P* = 0.54), average meal size (*P* = 0.26), eating time per day (*P* = 0.32), eating rate (*P* = 0.53), average meal length (*P* = 0.30), or intermeal interval (*P* = 0.84; Figure 7B–D).

### Daily Rhythms of Plasma Metabolites and Hormones

**Figure 8.**
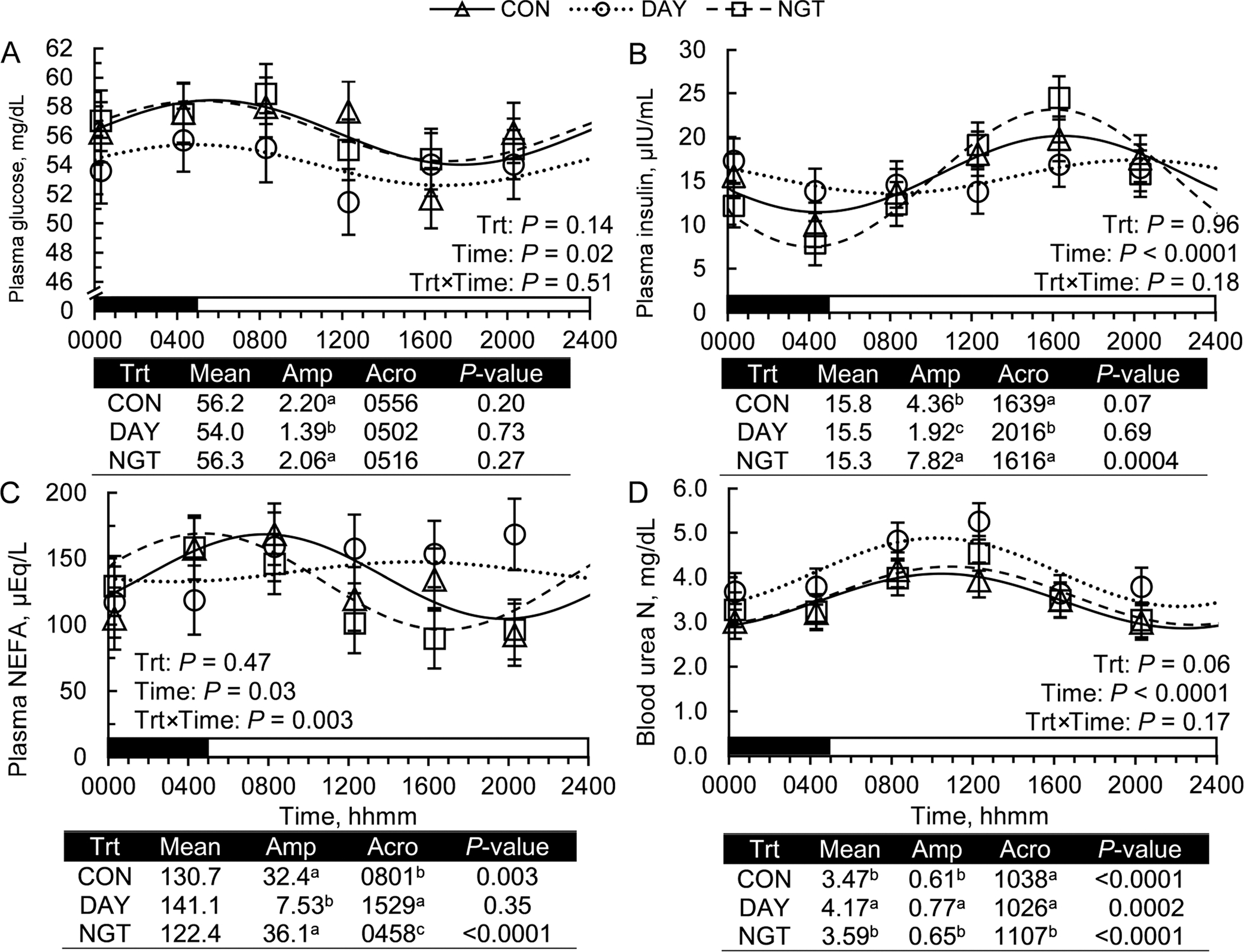
Effects of the timing of fatty acid (FA) infusion on daily rhythms of plasma metabolic parameters. Treatments were 350 g/d of free FA abomasally infused continuously for 22 h/d (CON), or for 8 h/d during the day (DAY; 0900–1700 h) or the night (NGT; 2100–0500 h). Panels include the effects of time of FA infusion on daily rhythms of plasma (A) glucose, (B) insulin, (C) nonesterified FA (NEFA), and (D) urea nitrogen concentrations. Data are presented as relative expression LSM with SEM bars and a fitted cosine curve. Output from cosinor analysis is shown below each panel and includes the amplitude (Amp; difference between peak and mean), acrophase (Acro; time at peak of the rhythm), and *P*-value of the zero-amplitude test. The black and white bars above the x-axis display the light:dark cycle. Means that do not share a superscript differ (*P* < 0.05).

Treatments did not affect average plasma glucose concentration (*P* = 0.14). Moreover, plasma glucose concentration did not show a 24-h rhythm in any treatment, as determined by the zero-amplitude test and cosinor analysis (*P* > 0.20; Figure 8A). Plasma insulin concentration similarly was not affected by treatment (*P* = 0.96), but a 24-h rhythm was present in NGT (*P* = 0.0004) and tended to be present during continuous FA infusion (*P* = 0.07; Figure 8B). Nighttime infusion of FA increased the amplitude of the daily rhythm of insulin concentrations compared with CON, whereas DAY decreased the amplitude of insulin concentration (*P* < 0.05).

Average plasma NEFA concentration was not affected by treatment (*P* = 0.47; Figure 8C). A 24-h rhythm in NEFA concentration was present in CON (*P* = 0.03) and NGT (*P* < 0.0001) but not DAY. The amplitude of the NEFA concentration rhythm did not differ between CON and NGT (*P* < 0.05), but was reduced in DAY. Additionally, DAY shifted the acrophase of the daily rhythm 7.5 h earlier, and NGT shifted the acrophase 5 h earlier, compared with CON (*P* < 0.05). Average BUN concentration was increased by DAY (*P* = 0.02), but not NGT (*P* = 0.67), compared with CON. Additionally, BUN concentrations fit a 24-h rhythm in all treatments (*P* < 0.0002). The amplitude of the daily rhythm of BUN was increased by DAY (*P* < 0.05) but not NGT (*P* > 0.10). The acrophase of BUN was also delayed by approximately 30 min during NGT but was not affected by DAY.

### Daily Rhythm of Body Temperature

**Figure 9.**
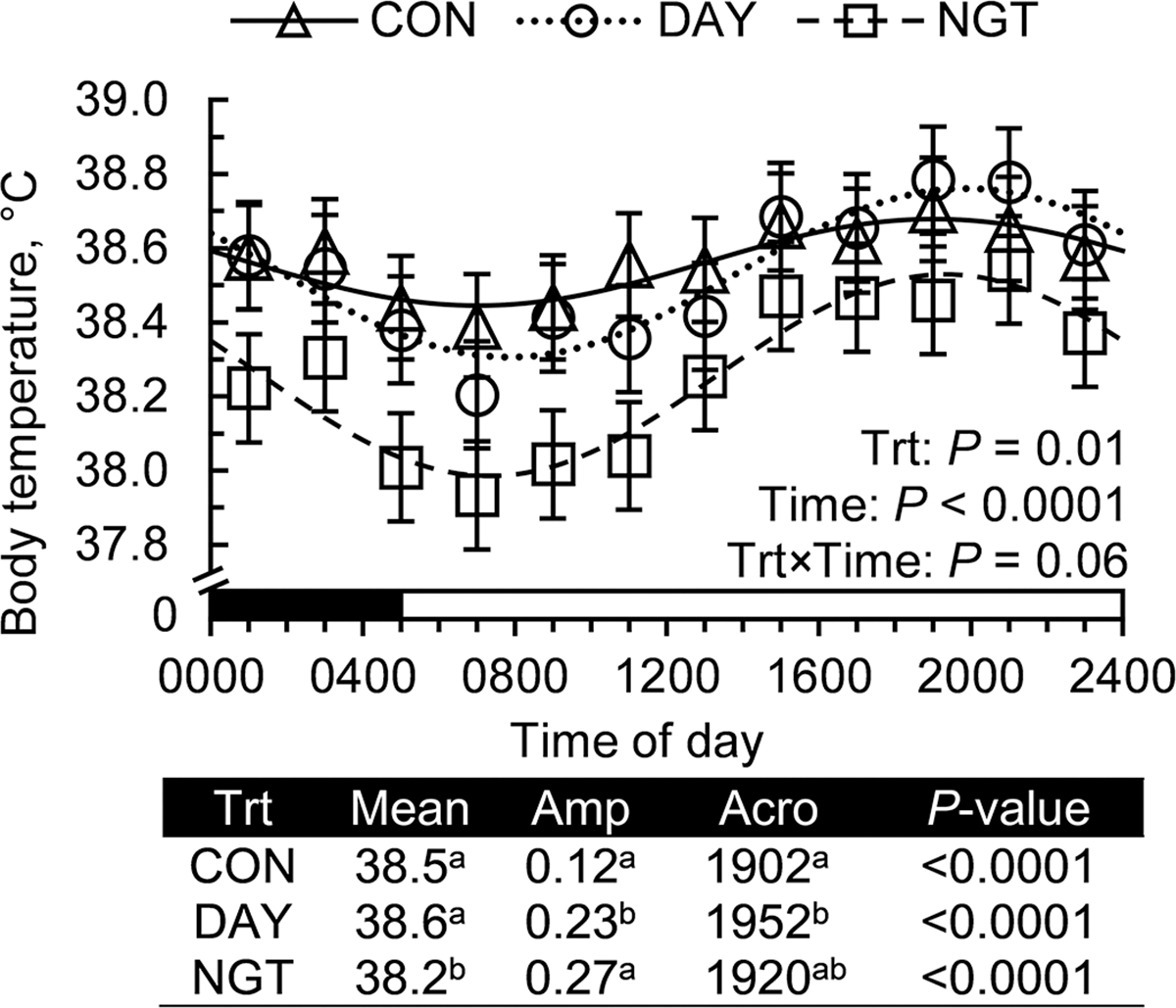
Effects of the timing of fatty acid (FA) infusions on the daily rhythms of core body temperature. Treatments were 350 g/d of free FA abomasally infused continuously for 22 h/d (CON), or for 8 h/d during the day (DAY; 0900–1700 h) or the night (NGT; 2100–0500 h). Data were collected every 10 min by a vaginal temperature data logger, averaged every 2 h, and presented as means and SEM with a fitted cosine curve. Output from cosinor analysis is shown and includes the amplitude (Amp; difference between peak and mean), acrophase (Acro; time at peak of the rhythm), and *P*-value of the zero-amplitude test. Means that do not share a superscript differ (*P* < 0.05). The black and white bars above the x-axis display the light:dark cycle.

The timing of FA infusion altered average body temperature (*P* = 0.02), with NGT decreasing body temperature by 0.3 and 0.4°C compared with CON and DAY, respectively (Figure 9). Body temperature fit a 24-h rhythm in all treatments (*P* < 0.0001), and the rhythm was altered by treatment. The amplitude of the daily rhythm was increased 92% by DAY and 125% by NGT (*P* < 0.05). Furthermore, the phase of the daily rhythm of body temperature was delayed 50 min by DAY but was not affected by NGT.

## DISCUSSION

The decrease in milk yield when the FA infusion was limited to either daytime or nighttime was not expected. The mechanism by which day- and night-restricted infusions of FA reduce these parameters is not clear. The simultaneous decrease in both milk and milk protein yields, but not milk fat yield, may indicate involvement of hormonal signals that regulate both lactose and protein, with less influence on milk fat. It is important to note that there were no differences in total DMI or eating behavior among treatments, indicating there were likely no differences in substrate intake. de Souza et al. (2021) reported that a calcium salt containing oleic acid increased body weight gain compared with a high palmitic acid supplement in early lactation. The higher rate of oleic acid infusion in the DAY and NGT treatments may have stimulated physiological mechanisms partitioning nutrients to body tissue gain, but the short treatment periods precluded investigating this. Oleic acid linearly increased insulin when infused as a bolus 4 times per day and may also affect insulin responsiveness (Prom et al., 2021). It is interesting that NGT infusion increased the amplitude of plasma insulin concentration with a higher peak during the day, when the infusion was not occurring, and DAY infusion decreased the amplitude of the rhythm by both increasing insulin at night and decreasing insulin during the day. A short delay from abomasal infusion to plasma appearance is expected, but these changes do not support a direct stimulation of insulin by oleic acid and may indicate other physiological adaptations such as changing the responsiveness of insulin release in the pancreas. The differences we observed in milk component synthesis and circulating metabolite concentrations could also be partially due to circadian differences in blood flow. Although not characterized in the mammary gland or in dairy cows, several factors controlling systemic blood flow, including cardiac output, vascular resistance, and glomerular filtration rate, have been shown to exhibit circadian rhythms in other species (Ayyar and Sukumaran, 2021).

The daily rhythms of milk yield and milk components are responsive to the timing of feed intake. In previous work, restricting the time of feed availability to 16 h, from 0700 to 2300 h, delayed the phase of the daily rhythm of milk yield by 8 h and advanced the phase of the rhythms of milk fat and protein concentration by 10 h and 14 h, respectively (Salfer and Harvatine, 2020). Furthermore, increasing the frequency of feeding from 1×/d to 4×/d delayed the peak of milk yield by 3 h and reduced the amplitude of the rhythms of fat and protein concentrations (Rottman et al., 2014). Previous work also suggests that individual nutrients can also influence the daily rhythms of milk synthesis. Using a similar design to this study, we have observed that postruminal infusions of sodium caseinate for 8 h during the day shifted the phase of the rhythm of milk fat yield approximately 6 h earlier and increased the amplitude of the rhythm nearly 4-fold compared with infusing the same quantity of sodium caseinate continuously throughout the day (Salfer et al., 2023). The increase in amplitude was associated with a 6% greater daily yield of milk fat. Additionally, limiting the time of ruminal infusion of sodium acetate to 8 h during the day increased the amplitudes of milk yield and milk fat and protein concentrations compared with continuous infusion throughout the day (Matamoros et al., 2022).

In the current experiment, phase shifts in the daily rhythms of fat and protein yields and concentrations were observed, but these effects were small (less than 3 h) and likely of little biological significance. In contrast, altering the timing of restricted feeding by 8 h caused an approximately 8-h shift in the phases of milk and protein yields and fat and protein concentrations (Salfer and Harvatine, 2020). The largest effects of FA infusions on the daily rhythms of milk synthesis were observed in the amplitudes of these rhythms. Fatty acid infusion during the daytime increased the amplitudes of milk, fat, and protein yields while reducing the amplitudes of fat and protein concentrations, compared with CON and NGT. These changes in amplitude may occur by altering amplitudes of canonical clock genes within the mammary gland. Dietary fat has been shown to reduce the amplitude of the daily rhythms of canonical clock genes as well as the clock-controlled genes that mediate FA synthesis in the adipose tissue and livers of mice (Kohsaka et al., 2007).

Although total daily FA yield was not reduced, a decrease was observed in daily production of de novo and mixed-origin FA in milk in both DAY and NGT groups. These results suggest that the higher infusion rate over the shorter duration of time may have reduced de novo FA synthesis in the mammary gland regardless of the time in which the infusion was given. Notably, no difference occurred in total >16 C FA yield across the day, but the amplitude was modified by DAY and NGT infusion. We observed a notable decrease in de novo and mixed-origin FA by 9% in both DAY and NGT, with a large decrease amplitude of the daily rhythm by NGT. Salfer and Harvatine (2020) observed that restricting feed intake to 16 h during the night decreased de novo and mixed FA yields relative to 16-h feed restriction during the day. However, this effect was not observed when only the time of feeding was altered and without restricting feed availability (Niu et al., 2014). Furthermore, Rottman et al. (2014) observed an increase in de novo synthesized and mixed-source FA produced per day when cows were fed 4×/d versus 1×/d.

Odd- and branched-chain FA are synthesized by microbes in the rumen or in the mammary gland from odd- and branched-chain VFA (Vlaeminck et al., 2006). As the FA used in our experiment were infused postruminally, the effects on milk OBCFA and OCFA concentrations due to treatment are surprising, because changes in FA type due to fermentation in the rumen would not be expected. Additionally, because neither daily DMI nor daily pattern of feed intake were affected by treatment, the observed results are not because of differences in the amount of fermentable DM entering the rumen. De novo synthesis of OBCFA can occur in the mammary gland via elongation of odd- and branched-chain VFA that are derived from ruminal fermentation (Fievez et al., 2012). Infusing FA during day- or nighttime may inhibit the elongation of propionyl-CoA into OCFA, thereby reducing total OBCFA concentration. Altering the timing of intake through time-restricted feeding does not alter OBCFA yields (Salfer and Harvatine, 2018, 2020). Consequently, our results seem to be specific to the timing of FA infusion. One possible cause for the reduced OBCFA in the DAY and NGT groups is that the faster rate of exogenous FA availability in these treatments may result in greater competition for FA absorption, thus inhibiting uptake of these rumen-derived FA.

Plasma glucose, insulin, and NEFA concentrations have previously been shown to exhibit a daily rhythm in cows (Giannetto and Piccione, 2009; Niu et al., 2014). When cows are fed in the morning, plasma glucose and NEFA levels peak in the early morning, with insulin levels peaking in the evening. However, feeding cows at night causes a phase inversion of the rhythms, with glucose and NEFA levels peaking in the evening and insulin levels peaking in the morning (Niu et al., 2014; Salfer and Harvatine, 2020). Removing access to feed during the middle of the day (between 1100 and 1900 h) also greatly increases the amplitude of the rhythms of NEFA concentration (Salfer and Harvatine, 2020).

In the current study, plasma glucose concentration was not rhythmic when FA infusions were limited to any period during the day. These results suggest that high doses of FA infused into the abomasum may dampen the rhythm of plasma glucose concentration. However, no negative control was included within this experiment to directly compare FA infusion against animals with no FA infusion. Despite the failure to fit a zero-amplitude test, cosinor rhythmometry demonstrated that the peak of the rhythm of plasma glucose concentrations occurred in the early morning in all treatments, consistent with previous characterizations of the plasma glucose rhythm in dairy cows fed in the morning (Niu et al., 2014; Salfer and Harvatine, 2020).

We previously observed that daytime infusion of protein increases the amplitude of plasma insulin concentration (Salfer et al., 2023). However, in the current FA infusion experiment, the opposite effect was observed, with daytime infusion dampening the rhythm of insulin concentrations. Infusing FA during the day had a similar effect on plasma NEFA concentration, reducing its amplitude relative to CON and NGT pattern. These results are consistent with insulin’s role in inhibiting lipolysis and controlling the release of NEFA into plasma (McNamara, 1995). Again, our FA results contrast with those observed during amino acid infusion, with daytime infusion of amino acids inducing a rhythm in NEFA concentrations, whereas no rhythm was observed during continuous infusion or nighttime infusion of amino acids (Salfer et al., 2023).

Regulation of glucose and adipose tissue metabolism by the molecular circadian clock has been demonstrated in many organisms. In mice, free FA concentrations in plasma exhibit daily rhythms that are under the direct control of the molecular circadian clock in adiopocytes (Shostak et al., 2013). Furthermore, lipid availability can entrain the molecular circadian clock of peripheral tissues through the peroxisome proliferator-activated receptor (PPAR) family of nuclear receptors. Minimal research has been conducted examining rhythms of circadian clock gene expression in bovine liver or adipose tissue; future research investigating the role of circadian rhythms in regulating the mechanisms of energy metabolism in cows is warranted.

**Figure 10.**
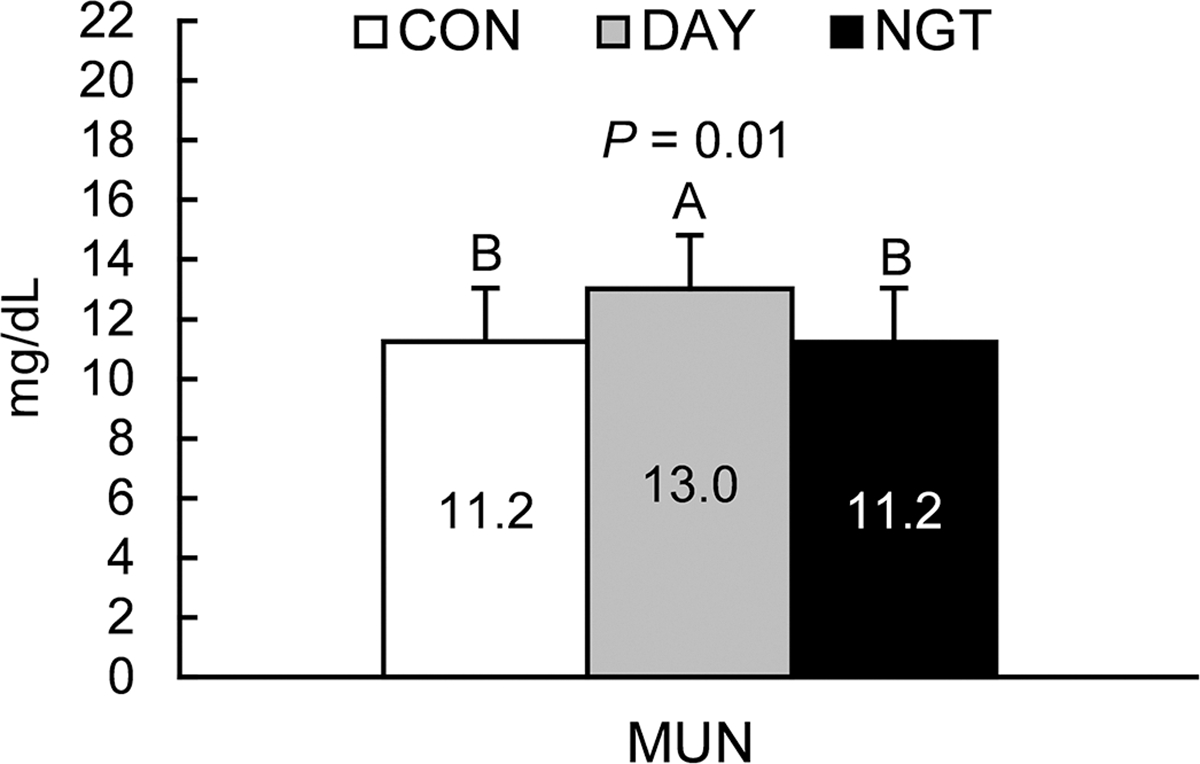
Effects of time of fatty acid (FA) infusions on MUN concentrations. Treatments were 350 g/d of free FA abomasally infused continuously for 22 h/d (CON), or for 8 h/d during the day (DAY; 0900–1700 h) or the night (NGT; 2100–0500 h). Data are presented as LSM with SEM bars. Means with differing uppercase letters (A, B) are different at *P* < 0.05.

As an indicator of nitrogen use efficiency, BUN is typically associated with increased ruminal ammonia concentrations (Jonker et al., 1998; Kohn et al., 2005). Consistent with our results, previous experiments demonstrate that BUN exhibits a highly robust daily rhythm that peaks in mid-morning (Giannetto and Piccione, 2009; Niu et al., 2017). We have previously demonstrated that limiting feeding time from 1900 to 1100 h inverts the rhythm of BUN (Salfer and Harvatine, 2020), and that abomasal infusions of amino acids from 0800 to 1700 h delays the phase of the BUN rhythm by approximately 6 h compared with continuous infusion (Salfer et al., 2023). In the current experiment, we observed minimal effects of the time of FA infusion on the daily rhythms of BUN. Daytime infusion, however, increased average daily BUN concentrations compared with CON and NGT. This result coincided with an increase in MUN in the DAY treatment (Figure 10). The differences in BUN and MUN in the DAY group may be due to the decreased milk protein yield in this treatment. Lower milk protein synthesis could lead to greater hepatic catabolism of amino acids, and greater BUN and subsequent MUN concentrations. The increased MUN could also be partially due to lower milk volume, thereby increasing the concentration of urea nitrogen in milk. Additional research focused on the timing of FA availability on mammary nutrient uptake and peripheral metabolism could help uncover these potential effects.

Previous research has suggested that altering the time of feed restriction dramatically shifts the phase of the daily rhythm of body temperature. We previously observed a ~9-h phase shift in the rhythm of body temperature when feed was restricted to 16 h in either the day or the nighttime (Salfer and Harvatine, 2020). Similar results were observed by Niu et al. (2014), who detected a 3-h shift in the phase of body temperature when cows were fed at 0830 versus 2030 h, without restricting feed availability. The results of the current study contrast with those of this previous work, and these discrepancies suggest that the time of FA absorption has little effect on the phasing of the rhythm of body temperature. Notably, the FA in the current experiment were delivered via abomasal infusion, and minimal differences occurred in the daily pattern of feed intake among treatments, resulting in negligible differences in diurnal patterns of nutrient composition across the day. This contrasts with experiments altering the time of feed delivery, which observed changes in the diurnal patterns of both feed intake and fecal indigestible NDF, indicating differences in daily changes in rumen passage. Rumen temperature oscillates in a circadian manner and is closely correlated with both DMI and core body temperature (Beatty et al., 2008; Piccione et al., 2014). The lack of a difference in the daily pattern of feed intake between treatments in our current study likely reduced any diurnal variations in rumen temperature, which may have contributed to the marginal differences in core body temperature that we observed.

## CONCLUSIONS

Daily rhythms of milk and milk component synthesis were altered by modifying the timing of FA infusion, with alterations in the rhythms’ amplitudes during daytime infusion. The lack of shifts in the acrophase of milk production and milk component rhythms suggests that changes in entrainment did not occur. Total daily milk yield was greatest during continuous infusion and was decreased during both day- and nighttime infusion, suggesting that a continuous supply of FA to the intestine is needed to maximize the rate of milk synthesis. The daily rhythms of insulin signaling is also affected by the timing of FA infusion, with daytime infusion dampening the rhythms of insulin and NEFA concentrations, perhaps through effects on the circadian clock of the pancreas. Future work should be conducted looking at the molecular mechanisms in the clocks of peripheral tissues that lead to alterations in the daily rhythms of hormones and metabolites.

## Figures and Tables

**Table 1.** Fatty acid profile of the free fatty acid (FA) stock used for abomasal infusions for study determining effects of the timing of FA infusion on the daily rhythms of milk synthesis and plasma hormones and metabolites in dairy cows

Name	g/100 g

14:0	0.22
15:0	0.01
16:0	5.11
*cis*-9 16:1	0.04
17:0	0.03
18:0	1.39
*trans*-9 18:1	0.26
*trans*-10 18:1	0.02
*trans*-11 18:1	0.38
*cis*-9 18:1	79.6
*cis*-11 18:1	0.56
*cis*-9, *cis*-12 18:2	10.0
*cis*-6, *cis*-9, *cis*-12 18:2	0.15
*cis*-9,*cis*-12,*cis*-15 18:2	0.36

**Table 2.** Diet and nutrient composition of the diet fed to all cows in a study determining the effects of the timing of abomasal infusion of fatty acids on the daily rhythms of milk synthesis and plasma hormones and metabolites in dairy cows

Item	Composition

Ingredients, % of DM	
Corn silage^1^	49.6
Alfalfa haylage^2^	15.0
Canola meal	12.5
Ground corn	11.6
Roasted soybeans	3.6
Grass hay/straw^3^	2.9
Mechanically-extracted soybean meal^4^	2.9
Vitamin/mineral mix^5^	1.9
Controlled-release N^6^	0.25
Nutrients, % of DM	
NDF	33.1
ADF	18.3
Starch	30.1
CP^7^	14.9
Ash	5.6

1 Contained (% of DM) 33.0% NDF and 16.9% ADF.

2 Contained (% of DM) 45.1% NDF and 34.7% ADF.

3 Contained (% of DM) 63.2% NDF and 33.5% ADF.

4 AminoPlus, Ag Processing Inc., Omaha, NE.

5 Contained (%, as-fed basis) 37.0% calcium carbonate, 29.9% dried corn distillers grains, 24.5% salt, 4.2% magnesium oxide (54% Mg), 2.4% organic phosphorus (15% P), 0.5% zinc sulfate, 0.2% mineral oil. Composition (DM basis): 7.2% CP, 7.1% NDF, 3.7% ADF, 15.0% Ca, 0.75% P, 0.33% K, 2.6% Mg, 0.5% S, 9.8% Na, 23.0 mg/kg Co, 652 mg/kg Cu, 783 mg/kg Fe, 54.0 mg/kg I, 1,190 mg/kg Mn, 12.8 mg/kg Se, 1,718 mg/kg Zn, 88,700 IU/kg vitamin A (retinyl acetate), 28,400 vitamin D (activated 7-dehydrocholesterol), 850 IU/kg vitamin E (dl-α tocopheryl acetate).

6 Fed as coated urea (Optigen, Alltech Inc., Lexington, KY; 259% CP, DM basis).

7 Estimated RDP of 73.0% of CP, based on NASEM, 2021.

## Nonstandard abbreviations used:

Acro: acrophase
Amp: amplitude
CON: 22-h continuous abomasal infusion of FA stock
DAY: daytime abomasal infusion of FA stock (8 h, from 0900 to 1700 h)
FA: fatty acid
NEFA: nonesterified fatty acid
NGT: nighttime abomasal infusion of FA stock (8 h, from 2100 to 0500 h)
OBCFA: odd- and branched-chain FA
OCFA: odd-chain FA
